# Smooth Muscle-Specific *BCL6+/−* Knockout Abrogates Sex Bias in Chronic Hypoxia-Induced Pulmonary Arterial Hypertension in Mice

**DOI:** 10.1155/2018/3473105

**Published:** 2018-07-24

**Authors:** Yang-Ming Yang, Pravin B. Sehgal

**Affiliations:** ^1^Department of Cell Biology and Anatomy, New York Medical College, Valhalla, NY 10595, USA; ^2^Department of Medicine, New York Medical College, Valhalla, NY 10595, USA

## Abstract

The “estrogen paradox” in pulmonary arterial hypertension (PAH) refers to observations that while there is a higher incidence of idiopathic PAH in women, rodent models of PAH show male dominance and estrogens are protective. To explain these differences, we previously proposed the neuroendocrine-STAT5-BCL6 hypothesis anchored in the sex-biased and species-specific patterns of growth hormone (GH) secretion by the pituitary, the targeting of the hypothalamus by estrogens to feminize GH secretion patterns, and the role of the transcription factors STAT5a/b and BCL6 as downstream mediators of this patterned GH-driven sex bias. As a test of this hypothesis, we previously reported that vascular smooth muscle cell- (SMC-) specific deletion of the *STAT5a/b* locus abrogated the male-dominant sex bias in the chronic hypoxia model of PAH in mice. In the present study, we confirmed reduced BCL6 expression in pulmonary arterial (PA) segments in both male and female SMC:*STAT5a/b−/−* mice. In order to test the proposed contribution of BCL6 to sex bias in PAH, we developed mice with SMC-specific deletion of *BCL6+/−* by crossing SM22*α*-Cre mice with *BCL6-floxed* mice and investigated sex bias in these mutant mice in the chronic hypoxia model of PAH. We observed that the male-bias observed in wild-type- (*wt*-) SM22*α*-*Cre-*positive mice was abrogated in the SMC:*BCL6+/−* knockouts—both males and females showed equivalent enhancement of indices of PAH. The new data confirm BCL6 as a contributor to the sex-bias phenotype observed in hypoxic PAH in mice and support the neuroendocrine-STAT5-BCL6 hypothesis of sex bias in this experimental model of vascular disease.

## 1. Introduction

Idiopathic pulmonary arterial hypertension (IPAH) is a disease with high morbidity and mortality with limited therapeutic options [1–5]. The histologic vascular lesions in the lungs are characterized by segmental pulmonary arterial remodeling and proliferation of the vascular smooth muscle and endothelial cells to form plexiform and “onion-skin” obliterative lesions which occlude the vascular lumen and cause increased mean pulmonary artery pressure (>25 mm Hg) and right ventricular hypertrophy [1–4]. Diverse inherited autosomal-dominant mutations in the *BMPR2* gene underlie a significant subset of IPAH cases, but with low penetrance [1–4, 6–8]. There is a distinct sexual dimorphism in IPAH in that this disease affects postpubertal women (median age at diagnosis: in the third decade) with 2–4-fold greater incidence than men (median age at diagnosis: in the fourth decade) [1–11]. However, disease progression is more rapid in men than in women [9]. The mechanisms that determine this sexual dimorphism in humans are incompletely understood. Puzzlingly, several studies have pointed to a direct protective effect of estrogens on development of an equivalent pulmonary arterial hypertension (PAH) disease process in experimental animals (such as in mice and rats exposed to chronic hypoxia or in rats exposed to monocrotaline pyrrole) ([9–21], and citations therein). Thus, in these experimental models in mice and rats it is the males that preferentially develop more pronounced disease, while exogenously administered estrogens have proven to be protective.

**Figure 1:**
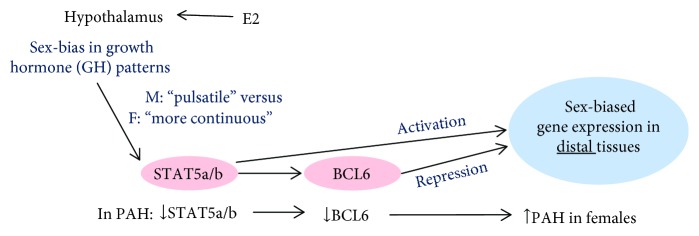
Schematic of the pathway involving the contributions of neuroendocrine-STAT5-BCL6 mechanisms of sex bias in PAH. Patterns of circulating GH in males typically consist of a low baseline level with few (2–4) but high peak levels (a pattern customarily referred to as “pulsatile”) [22–29]. Females have a higher baseline level of GH with small frequent peaks (≥7 per day) (a pattern referred to as “more continuous”) [22–29]. E2, estradiol-17*β*; M, male; F, female; downward short arrows indicate reduction; upward short arrow indicates increase.

In attempting to understand the mechanistic basis of this species-dependent sex-bias paradox (higher incidence of IPAH in women, but greater susceptibility to PAH in male mice and rats, with exogenous estrogens having a protective effect) [9–18], we discovered that smooth muscle cells (SMCs) in obliterative IPAH lesions in humans had a marked reduction in levels of the sex-responsive transcription factors STAT5a and STAT5b (collectively either “STAT5a/b” or “STAT5”), their activated Tyr-phosphorylated entities (PY-STAT5), and a downstream sex-responsive target of STAT5—the “broad-spectrum” transcriptional repressor B-cell lymphoma 6 protein (BCL6) (Figure 1) [19–21]. This discovery focused our attention on the possibility that the resolution of the species-specific sex-bias paradox in PAH between human females (greater susceptibility to PAH than males) and rodent females (lesser susceptibility to PAH than males) might reside in the well-known differences in the patterns of sex-biased secretion of growth hormone (GH)—a well-characterized upstream activator of STAT5a/b [19–21]. Women have a high baseline level of GH with small frequent peaks (a pattern referred to as “more continuous”), while men have a low baseline with few but high level peaks of GH (a pattern referred to as “pulsatile”) ([20–29], and citations therein) (Figure 1). Thus, circulating GH levels in men comprise 2–4 peaks per day with very low interpulse levels, while in women there is a higher frequency of pulses (>7 peaks/day) with significantly elevated interpulse levels (Figure 1) ([22–25]; reviewed in [20, 21]). Even when assayed at a single time-point in humans (in the morning after fasting), the median value of serum GH levels was 80–120-fold higher in young adult women than in young adult men [25]. This is a higher sexual dimorphism ratio than that for estradiol-17*β* (E2) (ratio: 2.2-fold female bias) or testosterone (ratio: 14-fold male bias) observed in the same sera [25]. Mice and rats also show similar quantitative differences in the respective male versus female patterns of circulating GH ([26–29], reviewed in [21]) (Figure 1). Male rodents show discrete GH pulses approximately every 3–5 hr with little or no circulating GH detectable during the interpulse interval, while in female rats, the pulses are more frequent, the pulse heights are lower, and the interpulse levels are relatively low but nonzero [26–29]. Overall, when compared to GH levels at peak pulse height, female humans have a relatively high interpulse level, while female rodents have a low interpulse level ([22–29]; reviewed in [20, 21]). We have pointed to this difference in relative interpulse levels as a key difference between women and female rodents that might underlie the estrogen paradox [19–21]. It is noteworthy that the pulsatility in GH levels is largely unconnected to the day-night circadian rhythms or the pulsatility of the cardiac cycle [20–29].

There is extensive neuroendocrinology literature showing that these patterns are derived from the patterned secretion of GH by the anterior pituitary in response to hypothalamic input via neuronal GHRH secretion which is in turn affected by estrogens (e.g., estradiol-17*β* (E2)) at the level of the arcuate nucleus (and other sites) in the ventromedial hypothalamus [30–42]. In terms of downstream mechanisms, there is also extensive literature mechanistically linking these GH pulsatility patterns to patterned activation of STAT5a/b (frequency and level of activation by Tyr phosphorylation) with resulting patterned activation of the transcriptional repressor BCL6 and hundreds of downstream genes (Figure 1) [43–51]. Remarkably, it has been established beginning in the late 1970s that the feminizing effect of exogenously administered estrogens on liver gene expression obligatorily required the pituitary in that hypophysectomy blocked this sex-bias effect of estrogens (Figure 1) [30–42]. Thus, although there is considerable research on the interactions between the estrogens, the estrogen receptors, and STAT5 and other transcription factors at the level of distal target tissues [47], there is also clear evidence that a major mechanism of the sex-bias effects of exogenously administered estrogens begins with the interaction of sex hormones with the hypothalamus and changes in the subsequent cascade of patterned activation of the GH-STAT5 axis in distal tissues (Figure 1) [30–51]. Thus, especially in light of the “estrogen paradox” in PAH [6, 9], we proposed the neuroendocrine-STAT5 hypothesis of sex bias in this disease to account for the difference between humans and rodents (Figure 1) [19–21].

Experimentally, after observing that SMCs in obstructive pulmonary arterial lesions in IPAH in both female and male patients showed reduced STAT5a/b and reduced PY-STAT5 [19], we tested the STAT5 hypothesis of sex bias in PAH in the chronic hypoxia model in mice. In this model, wild-type (*wt*) mice show a male-dominant phenotype. We developed tissue-specific knockout mice with heterozygous or homozygous deletion of *STAT5a/b* in vascular smooth muscle cells (SMCs) and investigated the influence of these deletions on the male dominance in this model of PAH [19]. We observed that SMC-specific heterozygous or homozygous deletion of *STAT5a/b* abrogated the male-dominant sex bias in that both female and male mice developed PAH (increased right ventricular systolic pressure (RVSP), hypertrophy of pulmonary arterial vessel wall, and right ventricular hypertrophy) to a similar extent [19–21].

We then focused on experimentally testing a downstream element in the GH-STAT5 hypothesis (Figure 1). It is well established that the transcriptional repressor BCL6 is one of the major transcription factors downstream of STAT5 in the sex-biased regulation of expression of hundreds of liver genes [50, 51]. We have previously reported that BCL6 was also reduced in SMCs in obliterative pulmonary arterial lesions in both women and men with IPAH concomitant with the reduction in STAT5a/b and PY-STAT5 levels [21]. It is also already known that whole-body homozygous deletion of *BCL6−/−* in the mouse led to extensive pulmonary edema and pulmonary parenchymal and vascular inflammation [52]. Thus, from the point of view of downstream mechanisms in the STAT5 hypothesis, we investigated (a) whether SMC-specific deletion of *STAT5a/b* in the mouse had an effect on expression of BCL6 in SMCs in pulmonary arterial tunica media and (b) whether SMC-specific deletion of *BCL6* in the mouse had an effect on chronic hypoxia-induced PAH and its male-dominant sex-bias phenotype (Figure 1). The new data reported here represent a successful test of the BCL6 element in the GH-STAT5-BCL6 hypothesis of sex bias in PAH [19–21].

## 2. Materials and Methods

### 2.1. Derivation of Mice with Smooth Muscle Cell- (SMC-) Specific Heterozygous and Homozygous Deletions of *STAT5a/b* or *BCL6*

Mouse breeding, care, and use of animals for these studies was approved by the Institutional Animal Care and Use Committee (IACUC) of the New York Medical College. A mouse line with SMC-specific homozygous deletion of the *STAT5a/b* locus had been previously produced by mating *STAT5a/b^fl/fl^* mice carrying *loxP* inserts bracketing the entire STAT5a/b locus in C57BL/6:129J mice provided by Dr. Lothar Hennighausen (National Institutes of Health, Bethesda, MD) with *SM22-Cre^+/+^* mice (expressing Cre recombinase under the control of the mouse transgelin (smooth muscle protein 22*α*) promoter) purchased from The Jackson Laboratories, Bar Harbor, ME (stock number: 004746 Tg (Tagln-cre)1Her/J in C57BL/6:129SJL background) [19]. PCR analyses of the tail DNA using appropriate primers were used to confirm the genotype of the *Cre*-positive mice with respect to the presence of *STAT5a/b wt* or *fl* or *Cre* alleles [19]. All mice in the present studies designated “*Wt*” were *Cre-*positive littermates not carrying any *STAT5a/b*-floxed alleles [19]. The selective reduction in STAT5 expression in the tunica media of pulmonary arterial segments in the *Cre-*positive heterozygous *STAT5a/b wt/fl* (designated “+/−”) or homozygous *fl/fl* (designated “−/−”) mice was verified in immunofluorescence and immunohistochemistry assays for STAT5 [19]. Smooth muscle *α-*actin- (SMA-) positive aortic smooth muscle cells (SMCs) derived from the homozygous knockouts showed a marked loss of STAT5a/b by Western blotting [19]. Lung tissues from *Wt* and SMC:*STAT5a/b−/−* mice used in the immunofluorescence analyses for BCL6 were from experiments previously reported in Figure 4 in [19].

**Figure 2:**
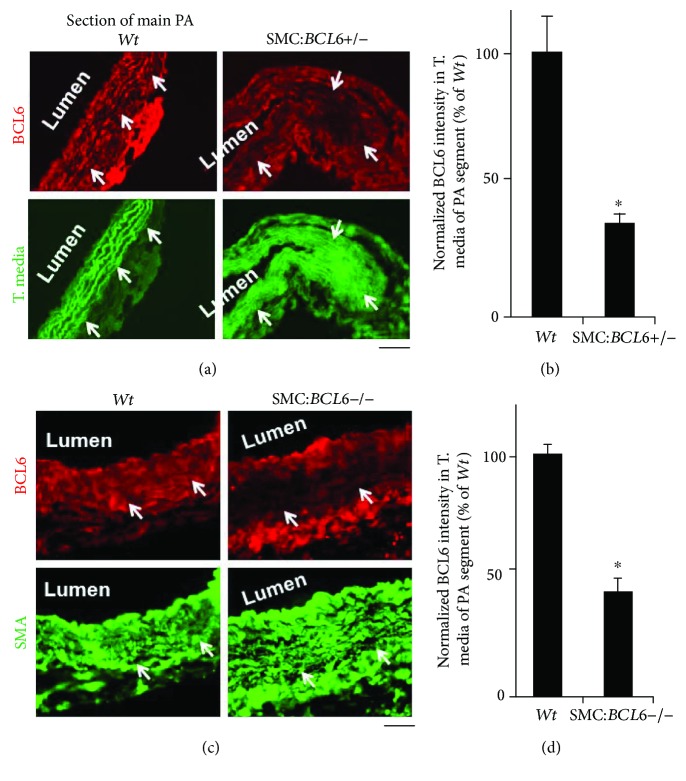
Reduced BCL6 expression in the tunica media/SMCs in sections of the main PA arteries in mice with SMC-specific heterozygous or homozygous deletion of the *BCL6* locus. Sections of the main PA arteries from *Wt* and respective mutant mice were evaluated for BCL6 expression by quantitative immunofluorescence. (a) Autofluorescence (in green) of the elastic laminae was used to identify the tunica media. (c) SMCs in the tunica media were immunostained for smooth muscle *α* actin (SMA). Arrows in the *Wt* sections draw attention to areas of normal BCL6 immunofluorescence and those in the mutant sections to areas with reduced BCL6 expression. (b, d) Summary of BCL6 immunofluorescence intensity in multiple areas of sections such as in (a) and (c) in the *Wt* and respective mutant mice. ^∗^*P* < 0.05 by Student's *t*-test.

Mouse lines (in C57BL/6 background) with SMC-specific heterozygous and homozygous deletions of *BCL6* were generated in the same manner described above by cross-mating the *BCL6^fl/fl^* (B6.129S (FVB)-*Bcl6^tm1.1Dent^*/J; stock number: 023727 Bcl6^fl^) and SM22*α*-Cre^+/+^ mice (stock number: 004746) purchased from The Jackson Laboratories, Bar Harbor, ME, and confirmed by PCR analyses of tail DNA using appropriate primers designated by the supplier. All mice in the BCL6 studies designated “*Wt*” were *Cre-*positive littermates not carrying any *BCL6*-floxed alleles. Reduced expression of BCL6 in the tunica media and SMCs of pulmonary artery segments of the mutant mice was confirmed by immunofluorescence analyses (Figure 2). These mutant mice, including the SMC-specific *STAT5a/b−/−* and SMC-specific *BCL6+/−* and −/− knockouts have no fertility or growth issues. In the present study, we used the more abundantly available male and female heterozygous SMC-specific *BCL6+/−* for the chronic hypoxia treatment.

### 2.2. Exposure to Hypoxia

Groups of *Wt*, or mutant male and female mice at approximately 8 weeks of age were used in the respective chronic hypoxia experiments (*n* = 4–7 mice per group per variable; average group size *n* = 5 mice) [19]. Mice were exposed to hypobaric hypoxia (0.5 atmospheres in room air equivalent to 10% *v*/*v* oxygen) for approximately 4 weeks; normoxia controls were left in ordinary room air [19].

### 2.3. Assessment of PAH and Lung Vascular Remodeling after Chronic Hypoxia

This was carried out as previously described in [19]. Briefly, mice were anaesthetized using the ketamine/xylazine cocktail (100 mg/kg ketamine, 10 mg/kg xylazine, intraperitoneally). A Millar catheter was used for the *in vivo* measurement of right ventricular systolic pressure (RVSP) via external jugular vein catheterization (PVR-1030; Millar Instruments Inc., Houston, TX). The animals were then euthanized and the heart, aorta, and left lung removed and formalin fixed (the right lung was snap-frozen in liquid N_2_). The heart was dissected to obtain the ratio of right ventricle weight to that of the left ventricle plus septum (RV/LV + S) as a measure of right ventricular hypertrophy (RVH). The left lung was used for paraffin block embedding and histology (hematoxylin & eosin, Van Gieson's elastin and Masson's Trichrome stains, and immunofluorescence for smooth muscle actin (SMA)). The extent of vascular remodeling in pulmonary arteries with an outer diameter < 80 *μ*m was quantitated in terms of medial wall thickness expressed as 2x wall thickness in % of average vessel outer diameter measured in two axes perpendicular to each other [19]. Muscularization of pulmonary vessels in lung sections was evaluated as indicated in Section 2.4. All histological quantitation was carried out in a blinded fashion.

### 2.4. Immunofluorescence Analyses of Lung Sections

Serial sections (5 *μ*m) of formalin-fixed, paraffin-embedded lung tissue were evaluated for BCL6 and smooth muscle *α*-actin (SMA) immunofluorescence as described earlier [19] using rabbit pAb for BCL6 or mouse mAb for SMA, and respective donkey AlexaFluor 594-tagged pAb (in red) as the secondary antibody. Imaging was carried out using a Zeiss AxioImager M2 motorized microscopy system equipped with a high-resolution RGB HRc AxioCam camera and AxioVision 4.8.1 software in a 1388 × 1040 pixel high-speed color capture mode. All data within each experiment were collected at identical imaging settings. Fluorescence intensity was quantitated using the NIH Image J software [19]. Muscularization of pulmonary vessels < 80 *μ*m in diameter was evaluated by immunofluorescence of SMA in lung sections and expressed as the mean number of SMA-positive vessels per 10x objective field.

### 2.5. Antibodies

Rabbit pAb to BCL6 (N-3) (Cat. number: sc-858) was purchased from Santa Cruz Inc., Santa Cruz, CA. Mouse mAb to SMA (Cat. number: A5228) was from Sigma-Aldrich Inc., St. Louis, MO. Alexa-Flour-tagged donkey secondary antibodies were from Life Technologies, Grand Island, NY.

### 2.6. Statistical Evaluations

These were performed using the two-tailed Student *t-*test (as in Figure 2) and single-factor ANOVA methods (NCSS version 8, 2012). Multiple group comparisons were carried out using ANOVA (Tukey-Kramer, Newman-Keuls, and Tukey-Kramer tests).

## 3. Results

### 3.1. SMC-Specific *STAT5a−/−* Deletion Leads to Reduced BCL6 Expression in Pulmonary Arterial Tunica Media

**Figure 3:**
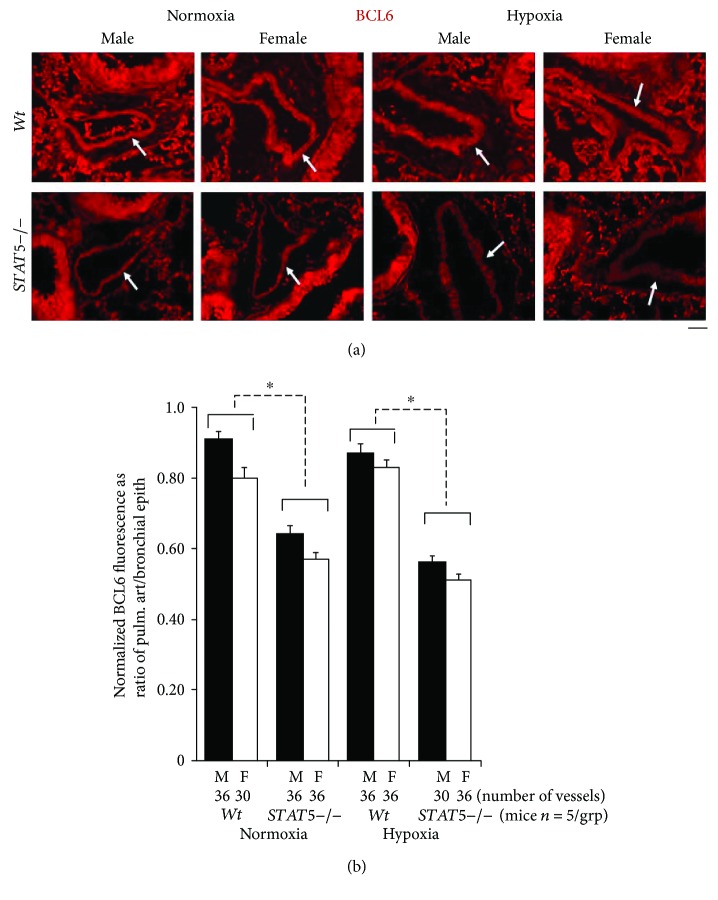
Reduced BCL6 expression in pulmonary arterial (PA) segments in SMC:*STAT5a/b−/−* male and female mice in normoxia and hypoxia. (a) Representative immunofluorescence images for expression of BCL6 in PA segments in lungs of *Wt* and SMC:*STAT5a/b−/−* male and female mice (arrows) kept for 4 weeks in normoxia or hypoxia. The lung sections used for this experiment derive from the mouse experiment reported in Figure 4 in [19]. Scale bar = 20 *μ*m. (b) Quantitation of BCL6 expression in PA segments as illustrated in (a) expressed as a ratio of fluorescence in PA segments compared to that in the adjacent bronchial epithelium in the same section. *n* = the number of arterial segments quantitated per group; ^∗^*P* < 0.05 by ANOVA.

We used quantitative immunofluorescence methods to investigate whether BCL6 might be downstream of STAT5 in SMCs in the pulmonary arterial vasculature. Groups of male and female *Wt* and *STAT5−/−* mice were exposed to hypoxia (10% hypobaric oxygen) for 4 weeks or kept under normoxic conditions, and the development of PAH was first evaluated as previously reported (RVSP, ratio of RV/LV + S weights, increased muscularization of vessels, and increased arterial wall thickness) [19]. From the data previously reported, we observed in this experiment that *STAT5−/−* deletion abrogated the male-dominant phenotype of PAH in this model—the knockout female and male mice developed equivalently high levels of PAH [19]. There was little change in heart rate or systemic blood pressure in these mice [19]. Subsequently, representative formalin-fixed paraffin-block sections of lungs were quantitatively evaluated for expression of BCL6 in the pulmonary arterial (PA) walls as a ratio to that in bronchial epithelium in different groups (Figure 3). The immunofluorescence data in Figure 3(a) (arrows) and the quantitation summarized in Figure 3(b) show a reduction in BCL6 immunofluorescence in PA segments in male and female *STAT5−/−* mice under both normoxic and hypoxic conditions. These data were consistent with the positioning of BCL6 downstream of STAT5 in mouse pulmonary arterial SMCs in a manner similar to its downstream position to STAT5 in the mouse liver (Figure 1) [49–51]. These data are consistent with the inference that STAT5a/b are positive transcription factors for expression of BCL6 in pulmonary arterial tissue (Figure 1), an inference previously validated extensively in the liver and isolated hepatocytes by Waxman and colleagues [47–51].

### 3.2. Development of SMC-Specific *BCL6+/−* and *BCL6−/−* Knockout Mice

We generated lines of C57Bl6 mice with heterozygous or homozygous deletions of the *BCL6* locus in vascular SMCs in crosses between *BCL6^fl/fl^* and *transgelin (SM22α)-Cre^+/+^* parents using methods previously described for the generation of SMC-specific *STAT5+/−* and −/− mice [19]. In addition to molecular characterization (using PCR methods summarized in [19]; data not shown), quantitative immunofluorescence methods were used to verify the reduced expression of BCL6 in the pulmonary arterial tunica media and SMCs in mice with SMC:*BCL6+/−* deletion (Figures 2(a) and 2(b)) and SMC:*BCL6−/−* deletion (Figures 2(c) and 2(d)). SMC-specific heterozygous and homozygous deletion of *BCL6* reduced the levels of expression of BCL6 in the tunica media/SMCs in PA segments of the respective knockout mice but not in the endothelium (Figures 2(a) and 2(c)).

### 3.3. Abrogation of the Male-Dominant Sex Bias in Hypoxic PAH in *BCL6+/−* Mice

**Figure 4:**
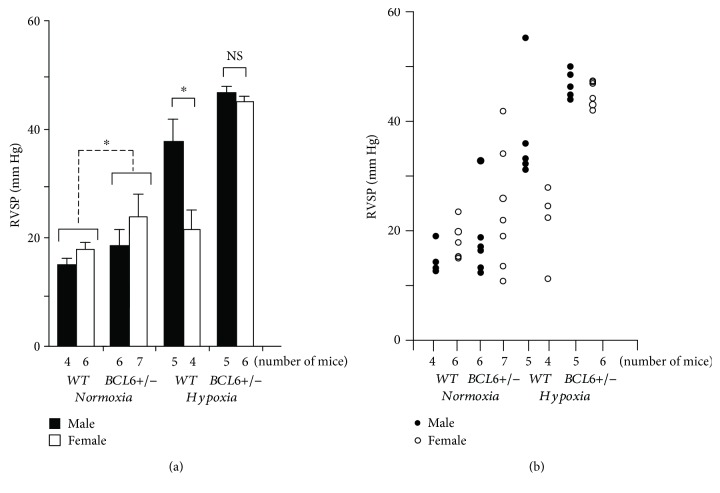
Abrogation of the male sex bias in chronic hypoxia-induced PAH in SMC:*BCL6+/−* mice: right ventricular systolic pressures (RVSP). Groups of male and female *Wt* and SMC:*BCL6+/−* knockout mice (*n* = 4–7 mice per group as indicated) were subjected to hypobaric hypoxia (10% *v*/*v* oxygen) or kept in room air for 4 weeks. At the end of the experiment, Millar catheterization was used to measure the RVSP (Figure 4 and [19]), the PA wall thickness (see Figure 5), muscularization of PA vessels in lung sections (Figure 6(a)), and right ventricular hypertrophy (the ratio of RV/LV + S wet weights) (Figure 6(b)). (a) Mean RVSP is shown in each group (mean ± SE). (b) The RVSP for each individual mouse is shown.^∗^*P* < 0.05 by ANOVA; NS, not significant.

We were able to generate sufficient SMC:*BCL6+/−* mice and *Cre-*positive “*wt*” littermates of both sexes to provide groups of 4–7 mice for evaluation indices of PAH per variable as indicated in Figure 4. (Only a limited number of SMC:*BCL6−/−* mice were generated in this project; these provided confirmatory preliminary data; see below.) Male and female *Wt* and SMC:*BCL6+/−* mice (*n* = 4–7 per group) were exposed to 10% hypobaric oxygen or normoxia for 4 weeks and evaluated for development of PAH. The data in Figure 4(a) show the mean RVSP in these groups in normoxia and in response to chronic hypoxia. There is a clear male-dominant phenotype in the increase in mean RVSP in *Wt* mice in response to hypoxia. In contrast, this sex bias was absent in *BCL6+/−* mice; both males and females showed increased RVSP in response to hypoxia.  Figure 4(b) shows the RVSP data for individual mice. The data show that even under normoxia, one male and three female *BCL6+/−* mice had RVSP levels > 25 mm Hg. Moreover, all male and female *BCL6+/−* hypoxic mice had markedly elevated RVSPs (Figure 4(b)). Overall, the data in Figure 4 provide evidence showing that (a) even an heterozygous deletion of *BCL6* in vascular SMCs led to an enhancement of the chronic hypoxia-induced increase in RVSP irrespective of sex, and (b) that there was a detectable increase in RVSP even in male and female knockout mice under normoxic conditions (Figure 4(a)). In preliminary studies, male and female homozygous SMC:*BCL6−/−* mice also showed an equivalent enhancement of RVSP after chronic hypoxia (data not shown).

**Figure 5:**
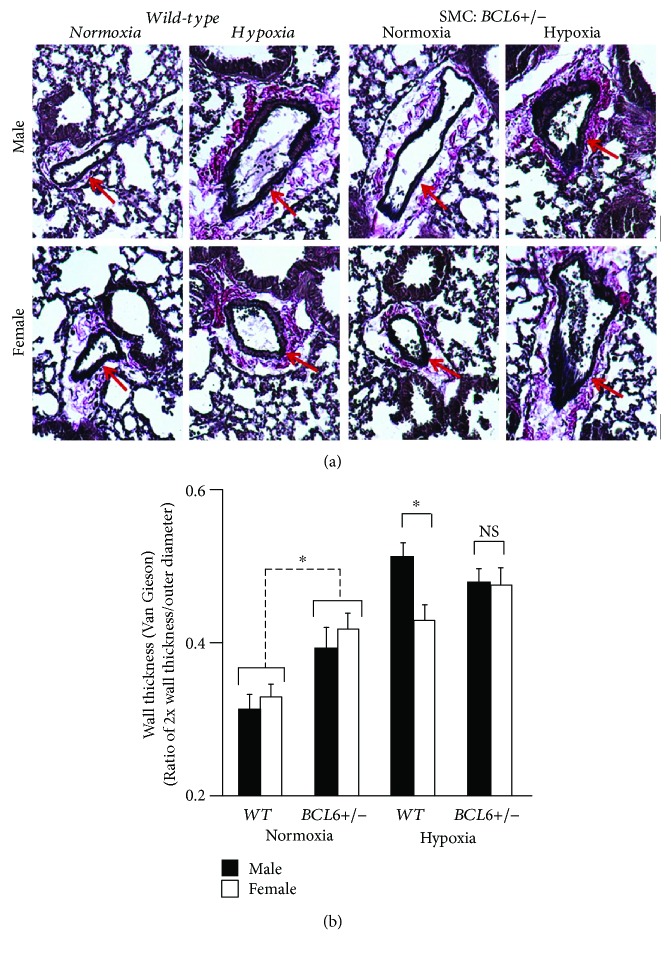
Abrogation of the male-sex bias in chronic hypoxia-induced PAH in SMC:*BCL6+/−* mice: hypertrophy of PA arterial wall thickness. Lungs derived from the experiment in Figure 4 were processed for histological sections and stained for elastin using Van Giesson's reagent. Wall thickness of PA segments with a diameter < 80 *μ*m was quantitated in terms of the ratio of 2x wall thickness/outer diameter as reported earlier [14]. (a) Representative histologic sections are illustrated. (b) The numerical data (mean ± SE) are summarized. Scale bar = 20 *μ*m. ^∗^*P* < 0.05 by ANOVA; NS, not significant.

**Figure 6:**
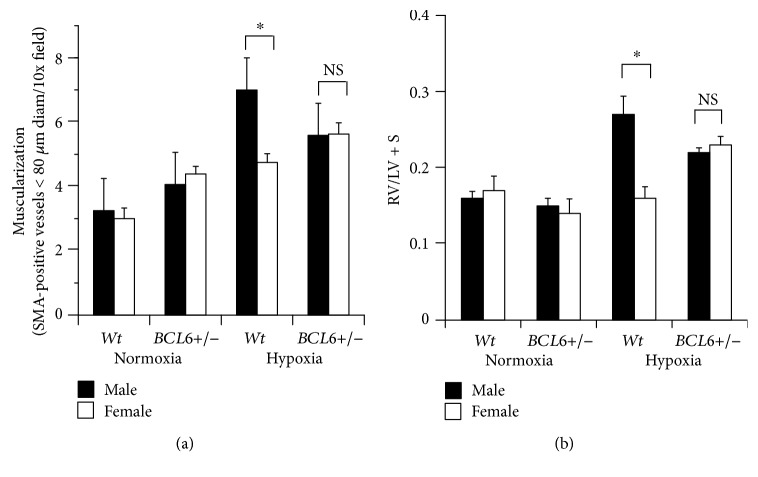
Abrogation of the male sex bias in chronic hypoxia-induced PAH in SMC:*BCL6+/−* mice: muscularization of pulmonary vessels in lung sections and right ventricular hypertrophy (Fulton's index). (a) Lung sections from mice shown in Figure 4 were immunostained for SMA and the average number of muscularized vessels per 10x field were enumerated for each (mean ± SE). (b) Hearts derived from the experiment in Figure 4 were fixed in 4% formalin, rinsed in PBS and dissected into the right ventricle (RV) and the left ventricle plus septum (LV + S) portions. The ratio of the wet weights of RV/LV + S (mean ± SE) illustrates the extent of right ventricular hypertrophy irrespective of the total weight of the individual heart. ^∗^*P* < 0.05 by ANOVA; NS, not significant.

Figures 5(a) and 5(b) summarize pulmonary arterial wall thickness data from the experiment in Figure 4. Again, the male-dominant sex bias in response to chronic hypoxia in terms of increased wall thickness in *Wt* mice was abrogated in *BCL6+/−* mice (Figures 5(a) and 5(b)). Moreover, there appeared to be an increase in wall thickness in both male and female knockout mice even under normoxic conditions (Figure 5(b)).  Figure 6(a) summarizes data pertaining to increased muscularization of pulmonary vessels in lung sections from hypoxic mice, while Figure 6(b) summarizes development of right ventricular hypertrophy measured in terms of the Fulton index (ratio of RV/LV + S weights). As expected, a male-dominant phenotype was observed in *Wt* hypoxic mice, but there was little sex bias in hypoxic SMC:*BCL6+/−* mice in terms of these indices of PAH (Figures 6(a) and 6(b)).

Taken together the data in Figures 4–6 provide evidence for a contribution of BCL6 in the sex-bias phenotype of PAH observed in chronically hypoxic mice. Even heterozygous deletion of *BCL6* in vascular SMCs rendered female mice equally susceptible to the development of hypoxic PAH as male mice. This observation was similar to our previous finding that heterozygous SMC-specific deletion of *STAT5a/b* was sufficient to abrogate the male-specific sex bias in the hypoxic mouse model [19].

## 4. Discussion

This study represents a specific test of the downstream BCL6 element in the GH-STAT5-BCL6 hypothesis of sex bias in PAH (Figure 1) [19–21]. In a previous study, we provided experimental support for this hypothesis by showing that vascular SMC-specific deletion of the *STAT5a/b* locus in mice abrogated the male-dominant sex bias observed in the chronic hypoxic PAH model [19]. In the present study, we have tested the next downstream element—BCL6—in this hypothesis (Figure 1). Consistent with its placement downstream of STAT5 [49–51], we observed reduced BCL6 levels in the tunica media/SMCs in pulmonary arterial segments in male and female mice with homozygous SMC-specific deletion of *STAT5a/b* (Figure 3). We then investigated whether SMC-specific deletion of *BCL6 (+/−)* affected the observed sex bias in the chronic hypoxia model. We observed the abrogation of the male-dominance in the development of PAH in the knockout mice. Both male and female SMC:*BCL6+/−* knockout mice responded equally to chronic hypoxia in terms of PAH development (Figures 4–6). Thus, these data place BCL6 in the STAT5-anchored sex-bias pathway in this experimental model and represent a successful critical test of the GH-STAT5-BCL6 hypothesis of sex bias in a vascular disease.

The novelty of the present study resides in the realization that insights accumulated over the last 30–40 years in a sister field (sex-biased expression of liver genes) and the neuroendocrine mechanisms by which exogenously administered estrogens influences these (Figure 1) [30–51] is largely missing from the vascular biology field, and especially the pulmonary hypertension field [1–18]. The critical insight from the liver field from the 1970s, was that the effects of exogenously administered estrogens on feminization of sex-biased gene expression in the liver in rodent models were absolutely dependent on the pituitary—hypophysectomy blocked the feminization (Figure 1) [30–42]. The target of injected estrogens included the arcuate nucleus, and additional nuclei, in the hypothalamus (Figure 1) [30–42]. In terms of vascular biology, that hypophysectomy in the male rat markedly impaired arterial remodeling after aortic balloon injury in terms of reduced vascular SMC proliferation and myointima formation, was reported in 1978 [53], and confirmed [54], but this research was not pursued further. Moreover, that ghrelin, a GH secretagogue and vasodilator (which antagonizes endothelin-1), inhibited hypoxia- or monocrotaline-induced PA remodeling in male rats is now known [55–57], yet neuroendocrine mechanisms have received little attention in the sex bias observed in vascular disease. Thus far, most research on sex bias in the vascular field has focused on the direct effects of sex hormones on vascular tissues [6, 7, 9–18].

As elucidated in detail by Waxman et al. and other investigators in the last two-three decades, this neuroendocrine mechanism of sex bias, operating through an axis consisting of the arcuate nucleus, growth hormone releasing hormone (GHRH), growth hormone (GH), and signal transducer and activator of transcription 5 (STAT5), accounts for sex-biased expression of ~500–1000 genes in the liver, and also of body growth and body weight (Figure 1) [43–51].

Downstream of STAT5 is a major cascade of sex-specific regulated gene expression of 500–1000 genes (including other transcription repressors such as BCL6; transcription activators such as Cux2, HNF4*α*, HNF6, and P450 CYP enzyme species; cell-cycle regulators; cytokines; growth factors; and immune mediators) [43–51]. As a foundational experiment in the liver field, Waxman et al. [43–46] observed that the conditional liver-specific deletion of the *STAT5a/b* locus in the mouse using the albumin-Cre and loxP approach led to the abrogation of sex-biased gene expression in the liver.

We studied STAT5a/b in vascular cells in terms of its contribution to the sex-bias phenotype in PAH induced in mice following chronic hypoxia [19–21]. We generated mice with heterozygous or homozygous conditional deletions of the *STAT5a/b* locus in vascular smooth muscle cells (SMCs). Although, *Wt* males subjected to hypoxic stress showed significant pulmonary arterial and cardiac remodeling, with *Wt* females showing minimal changes (a male-dominant phenotype), the SMC-specific *STAT5 −/−* mice hypoxic females showed the severest manifestations of PA remodeling (a female-dominant phenotype) [19]. The reversal of sex bias in this model of PAH did not require the complete loss of *STAT5a/b* genes. Even heterozygous conditional *STAT5a/b*+/− mice (thus, with a 50% loss of STAT5a/b) showed a loss of the male-dominant PAH phenotype [19]. The observation in the present study that these SMC:*STAT5a/b−/−* mice also showed reduced expression of BCL6 in the tunica media/SMCs in pulmonary arterial segments in both normoxic and hypoxic mice irrespective of sex (Figure 3) was consistent with the known role of STAT5 as a transcription factor activating BCL6 gene expression (Figure 1) [47–51]. In as much as it is known that BCL6 is a broad-spectrum transcriptional repressor [49–52], the reduced expression of BCL6 in SMC:*STAT5a/b−/−* mice prompted us to test the involvement of BCL6 per se in the sex bias observed in this model of PAH by genetically deleting *BCL6* in SMCs in mice. Since it has already been shown that mice with whole-body knockout of the transcriptional repressor BCL6 showed increased production of proinflammatory cytokines (IL-6, IFN-gamma, and IL-4), developed generalized vasculitis including pulmonary vasculitis, grew poorly, and lived only 6–8 weeks [52], in the present study we specifically investigated the effects of tissue-specific BCL6 deletion only in vascular SMCs.

We discovered that SMC-specific deletion of *BCL6* led to an abrogation of the male-dominant sex bias seen in hypoxic *Wt* mice. The data provide evidence for the involvement of the BCL6 axis in the sex bias observed in the chronic hypoxia-induced model of PAH in mice. This represents a successful test of the neuroendocrine-STAT5 hypothesis of sex bias in hypoxic PAH in mice in terms of the involvement of a predicted downstream player—the involvement of BCL6.

In preliminary experiments, we have initiated tests of the involvement of the predicted upstream participant—the pituitary—in sex bias in hypoxic PAH in mice (Figure 1). Preliminary data suggest that hypophysectomy also abrogates the sex bias observed in *Wt* mice (data not shown).

We have previously reported that a marked decrease in STAT5a/b and BCL6 was observed in SMCs in obliterative lesions in human IPAH—in women and men [19, 21]. These observations suggested a role of aberrations in the STAT5-BCL6 axis in the pathogenesis of the human disease. It is long established that 30–50% of patients with acromegaly develop systemic hypertension [58–60]. Although respiratory complications in acromegaly are known to include sleep-breathing disorders and “respiratory insufficiency” [60], there is little information about the pulmonary arterial circulation per se in such patients. Whether patients with IPAH have altered GH secretion kinetics could be evaluated using patterns of exercise-induced GH secretion [61–63].

## 5. Conclusions

The present study represents a successful test of one downstream element—BCL6—in the neuroendocrine-GH-STAT5-BCL6 sex-bias hypothesis in an experimental model of PAH. The insights obtained are likely to apply to sex bias in other vascular disease models and the human disease [20, 21]. While the present study has focused on the neuroendocrine pathway that indirectly mediates the feminizing effect of estrogens, we realize that there are extensive studies that highlight the direct effects of estrogens in vascular tissues [6, 7, 9–18]. From our perspective, the development of sexual dimorphism in vascular disease likely includes both upstream indirect effects initiated by sex hormones at the hypothalamus and mediated through the GH-STAT5-BCL6 pathway [19–21] as well as direct effects of sex hormones on vascular cells [6, 7, 9–18].

## Data Availability

The data used to support the findings of this study are included within the article.

## References

[1] Tuder R. M., Marecki J. C., Richter A., Fijalkowska I., Flores S. (2007). Pathology of pulmonary hypertension. *Clinics in Chest Medicine*.

[2] Rabinovitch M. (2008). Molecular pathogenesis of pulmonary arterial hypertension. *The Journal of Clinical Investigation*.

[3] Morrell N. W. (2010). Role of bone morphogenetic protein receptors in the development of pulmonary arterial hypertension. *Advances in Experimental Medicine and Biology*.

[4] Stacher E., Graham B. B., Hunt J. M. (2012). Modern age pathology of pulmonary arterial hypertension. *American Journal of Respiratory and Critical Care Medicine*.

[5] Hensley M. K., Levine A., Gladwin M. T., Lai Y. C. (2018). Emerging therapeutics in pulmonary hypertension. *American Journal of Physiology-Lung Cellular and Molecular Physiology*.

[6] Austin E. D., Cogan J. D., West J. D. (2009). Alterations in oestrogen metabolism: implications for higher penetrance of familial pulmonary arterial hypertension in females. *European Respiratory Journal*.

[7] Austin E. D., Hamid R., Hemnes A. R. (2012). BMPR2 expression is suppressed by signaling through the estrogen receptor. *Biology of Sex Differences*.

[8] Fessel J. P., Loyd J. E., Austin E. D. (2011). The genetics of pulmonary arterial hypertension in the post-*BMPR2* era. *Pulmonary Circulation*.

[9] Lahm T., Tuder R. M., Petrache I. (2014). Progress in solving the sex hormone paradox in pulmonary hypertension. *American Journal of Physiology-Lung Cellular and Molecular Physiology*.

[10] Mair K. M., Johansen A. K. Z., Wright A. F., Wallace E., MacLean M. R. (2014). Pulmonary arterial hypertension: basis of sex differences in incidence and treatment response. *British Journal of Pharmacology*.

[11] Umar S., Rabinovitch M., Eghbali M. (2012). Estrogen paradox in pulmonary hypertension—current controversies and future perspectives. *American Journal of Respiratory and Critical Care Medicine*.

[12] Liu A., Schreier D., Tian L. (2014). Direct and indirect protection of right ventricular function by estrogen in an experimental model of pulmonary arterial hypertension. *American Journal of Physiology-Heart and Circulatory Physiology*.

[13] Frump A. L., Goss K. N., Vayl A. (2015). Estradiol improves right ventricular function in rats with severe angioproliferative pulmonary hypertension: effects of endogenous and exogenous sex hormones. *American Journal of Physiology-Lung Cellular and Molecular Physiology*.

[14] White K., Dempsie Y., Nilsen M., Wright A. F., Loughlin L., MacLean M. R. (2011). The serotonin transporter, gender, and 17*β* oestradiol in the development of pulmonary arterial hypertension. *Cardiovascular Research*.

[15] Dempsie Y., Nilsen M., White K. (2011). Development of pulmonary arterial hypertension in mice over-expressing S100A4/Mts1 is specific to females. *Respiratory Research*.

[16] Fessel J. P., Chen X., Frump A. (2013). Interaction between bone morphogenetic protein receptor type 2 and estrogenic compounds in pulmonary arterial hypertension. *Pulmonary Circulation*.

[17] Mair K. M., Yang X. D., Long L. (2015). Sex affects bone morphogenetic protein type II receptor signaling in pulmonary artery smooth muscle cells. *American Journal of Respiratory and Critical Care Medicine*.

[18] Wright A. F., Ewart M. A., Mair K. (2015). Oestrogen receptor alpha in pulmonary hypertension. *Cardiovascular Research*.

[19] Yang Y. M., Yuan H., Edwards J. G. (2015). Deletion of *STAT5a/b* in vascular smooth muscle abrogates the male bias in hypoxic pulmonary hypertension in mice: implications in the human disease. *Molecular Medicine*.

[20] Sehgal P. B., Yang Y. M., Miller E. J. (2015). Hypothesis: neuroendocrine mechanisms (hypothalamus-growth hormone-STAT5 axis) contribute to sex bias in pulmonary hypertension. *Molecular Medicine*.

[21] Sehgal P. B., Yang Y. M., Yuan H., Miller E. J. (2015). STAT5a/b contribute to sex bias in vascular disease: a neuroendocrine perspective. *JAK-STAT*.

[22] Winer L. M., Shaw M. A., Baumann G. (1990). Basal plasma growth hormone levels in man: new evidence for rhythmicity of growth hormone secretion. *The Journal of Clinical Endocrinology & Metabolism*.

[23] van den Berg G., Veldhuis J. D., Frolich M., Roelfsema F. (1996). An amplitude-specific divergence in the pulsatile mode of growth hormone (GH) secretion underlies the gender difference in mean GH concentrations in men and premenopausal women. *The Journal of Clinical Endocrinology & Metabolism*.

[24] Pincus S. M., Gevers E. F., Robinson I. C. (1996). Females secrete growth hormone with more process irregularity than males in both humans and rats. *American Journal of Physiology-Endocrinology and Metabolism*.

[25] Engström B. E., Karlsson F. A., Wide L. (1998). Marked gender differences in ambulatory morning growth hormone values in young adults. *Clinical Chemistry*.

[26] Edén S. (1979). Age- and sex-related differences in episodic growth hormone secretion in the rat. *Endocrinology*.

[27] MacLeod J. N., Pampori N. A., Shapiro B. H. (1991). Sex differences in the ultradian pattern of plasma growth hormone concentrations in mice. *Journal of Endocrinology*.

[28] Painson J. C., Tannenbaum G. S. (1991). Sexual dimorphism of somatostatin and growth hormone-releasing factor signaling in the control of pulsatile growth hormone secretion in the rat. *Endocrinology*.

[29] Dhir R. N., Shapiro B. H. (2003). Interpulse growth hormone secretion in the episodic plasma profile causes the sex reversal of cytochrome P450s in senescent male rats. *Proceedings of the National Academy of Sciences of the United States of America*.

[30] Colby H. D., Gaskin J. H., Kitay J. I. (1973). Requirement of the pituitary gland for gonadal hormone effects on hepatic corticosteroid metabolism in rats and hamsters. *Endocrinology*.

[31] Kramer R. E., Greiner J. W., Rumbaugh R. C., Sweeney T. D., Colby H. D. (1979). Requirement of the pituitary gland for gonadal hormone effects on hepatic drug metabolism in rats. *Journal of Pharmacology and Experimental Therapeutics*.

[32] Rumbaugh R. C., Colby H. D. (1980). Is growth hormone the pituitary feminizing factor mediating the actions of estradiol on hepatic drug and steroid metabolism?. *Endocrinology*.

[33] Sakuma T., Endo Y., Mashino M. (2002). Regulation of the expression of two female-predominant CYP3A mRNAs (CYP3A41 and CYP3A44) in mouse liver by sex and growth hormones. *Archives of Biochemistry and Biophysics*.

[34] Nishida Y., Yoshioka M., St-Amand J. (2005). Sexually dimorphic gene expression in the hypothalamus, pituitary gland, and cortex. *Genomics*.

[35] Low M. J., Otero-Corchon V., Parlow A. F. (2001). Somatostatin is required for masculinization of growth hormone-regulated hepatic gene expression but not of somatic growth. *The Journal of Clinical Investigation*.

[36] Desjardins G. C., Brawer J. R., Beaudet A. (1993). Estradiol is selectively neurotoxic to hypothalamic beta-endorphin neurons. *Endocrinology*.

[37] Brawer J. R., Beaudet A., Desjardins G. C., Schipper H. M. (1993). Pathologic effect of estradiol on the hypothalamus. *Biology of Reproduction*.

[38] Kelly M. J., Ronnekleiv O. K. (2015). Minireview: neural signaling of estradiol in the hypothalamus. *Molecular Endocrinology*.

[39] Muller E. E., Locatelli V., Cocchi D. (1999). Neuroendocrine control of growth hormone secretion. *Physiological Reviews*.

[40] Garcia-Segura L. M., Baetens D., Naftolin F. (1986). Synaptic remodelling in arcuate nucleus after injection of estradiol valerate in adult female rats. *Brain Research*.

[41] Shirasu K., Stumpf W. E., Madhabananda SAR (1990). Evidence for direct action of estradiol on growth hormone-releasing factor (GRF) in rat hypothalamus: localization of (3H)estradiol in GRF neurons. *Endocrinology*.

[42] Señaris R. M., Lago F., Lewis M. D., Dominguez F., Scanlon M. F., Dieguez C. (1992). Differential effects of in vivo estrogen administration on hypothalamic growth hormone releasing hormone and somatostatin gene expression. *Neuroscience Letters*.

[43] Waxman D. J., Ram P. A., Park S. H., Choi H. K. (1995). Intermittent plasma growth hormone triggers tyrosine phosphorylation and nuclear translocation of a liver-expressed, Stat 5-related DNA binding protein: proposed role as an intracellular regulator of male-specific liver gene transcription. *Journal of Biological Chemistry*.

[44] Gebert C. A., Park S. H., Waxman D. J. (1997). Regulation of signal transducer and activator of transcription (STAT) 5b activation by the temporal pattern of growth hormone stimulation. *Molecular Endocrinology*.

[45] Tannenbaum G. S., Choi H. K., Gurd W., Waxman D. J. (2001). Temporal relationship between the sexually dimorphic spontaneous GH secretory profiles and hepatic STAT5 activity. *Endocrinology*.

[46] Udy G. B., Towers R. P., Snell R. G. (1997). Requirement of STAT5b for sexual dimorphism of body growth rates and liver gene expression. *Proceedings of the National Academy of Sciences of the United States of America*.

[47] Waxman D. J., Holloway M. G. (2009). Sex differences in the expression of hepatic drug metabolizing enzymes. *Molecular Pharmacology*.

[48] Holloway M. G., Cui Y., Laz E. V., Hosui A., Hennighausen L., Waxman D. J. (2007). Loss of sexually dimorphic liver gene expression upon hepatocyte-specific deletion of *Stat5a-Stat5b* locus. *Endocrinology*.

[49] Zhang Y., Laz E. V., Waxman D. J. (2012). Dynamic, sex-differential STAT5 and BCL6 binding to sex-biased, growth hormone-regulated genes in adult mouse liver. *Molecular and Cellular Biology*.

[50] Meyer R. D., Laz E. V., Su T., Waxman D. J. (2009). Male-specific hepatic Bcl6: growth hormone-induced block of transcription elongation in females and binding to target genes inversely coordinated with STAT5. *Molecular Endocrinology*.

[51] Sugathan A., Waxman D. J. (2013). Genome-wide analysis of chromatin states reveals distinct mechanisms of sex-dependent gene regulation in male and female mouse liver. *Molecular and Cellular Biology*.

[52] Nurieva R. I., Chung Y., Martinez G. J. (2009). BCL6 mediates the development of T follicular helper cells. *Science*.

[53] Tiell M. L., Stemerman M. B., Spaet T. H. (1978). The influence of the pituitary on arterial intimal proliferation in the rat. *Circulation Research*.

[54] Khorsandi M., Fagin J. A., Fishbein M. C., Forrester J. S., Cercek B. (1992). Effects of hypophysectomy on vascular insulin-like growth factor-I gene expression after balloon denudation in rats. *Atherosclerosis*.

[55] Henriques-Coelho T., Correia-Pinto J., Roncon-Albuquerque R. (2004). Endogenous production of ghrelin and beneficial effects of its exogenous administration in monocrotaline-induced pulmonary hypertension. *American Journal of Physiology-Heart and Circulatory Physiology*.

[56] Xu Y. P., Zhu J. J., Cheng F. (2011). Ghrelin ameliorates hypoxia-induced pulmonary hypertension via phospho-GSK3*β*/*β*-catenin signaling in neonatal rats. *Journal of Molecular Endocrinology*.

[57] Perchard R., Clayton P. E. (2017). Ghrelin and growth. *Endocrine Development*.

[58] Fedrizzi D., Rodrigues T. C., Costenaro F., Scalco R., Czepielewski M. A. (2011). Hypertension-related factors in patients with active and inactive acromegaly. *Arquivos Brasileiros de Endocrinologia & Metabologia*.

[59] Sardella C., Cappellani D., Urbani C. (2016). Disease activity and lifestyle influence comorbidities and cardiovascular events in patients with acromegaly. *European Journal of Endocrinology*.

[60] Pivonello R., Auriemma R. S., Grasso L. F. S. (2017). Complications of acromegaly: cardiovascular, respiratory and metabolic comorbidities. *Pituitary*.

[61] Kurtais Y., Tur B. S., Elhan A. H., Erdogan M. F., Yalcin P. (2006). Hypothalamic-pituitary-adrenal hormonal responses to exercise stress test in patients with rheumatoid arthritis compared to healthy controls. *The Journal of Rheumatology*.

[62] Zueger T., Alleman S., Christ E. R., Stettler C. (2011). Exercise-induced GH secretion in the assessment of GH deficiency in adult individuals. *European Journal of Endocrinology*.

[63] Eliakim A., Nemet D., Most G., Rakover N., Pantanowitz M., Meckel Y. (2014). Effect of gender on the GH-IGF-I response to anaerobic exercise in young adults. *Journal of Strength and Conditioning Research*.

